# Host Cell Proteins in Biologics Manufacturing: The Good, the Bad, and the Ugly

**DOI:** 10.3390/antib6030013

**Published:** 2017-09-16

**Authors:** Martin Kornecki, Fabian Mestmäcker, Steffen Zobel-Roos, Laura Heikaus de Figueiredo, Hartmut Schlüter, Jochen Strube

**Affiliations:** 1 Institute for Separation and Process Technology, Clausthal University of Technology, Leibnizstr. 15, 38678 Clausthal-Zellerfeld, Germany; kornecki@itv.tu-clausthal.de (M.K.); mestmaecker@itv.tu-clausthal.de (F.M.); zobel-roos@itv.tu-clausthal.de (S.Z.-R.); 2 Institute of Clinical Chemistry, Department for Mass Spectrometric Proteomics, University Medical Center Hamburg-Eppendorf, Martinistr. 52, 20246 Hamburg, Germany; l.heikaus@uke.de (L.H.d.F.); hschluet@uke.de (H.S.)

**Keywords:** upstream, downstream, host cell protein, CHO, ATPE, iCCC

## Abstract

Significant progress in the manufacturing of biopharmaceuticals has been made by increasing the overall titers in the USP (upstream processing) titers without raising the cost of the USP. In addition, the development of platform processes led to a higher process robustness. Despite or even due to those achievements, novel challenges are in sight. The higher upstream titers created more complex impurity profiles, both in mass and composition, demanding higher separation capacities and selectivity in downstream processing (DSP). This creates a major shift of costs from USP to DSP. In order to solve this issue, USP and DSP integration approaches can be developed and used for overall process optimization. This study focuses on the characterization and classification of host cell proteins (HCPs) in each unit operation of the DSP (i.e., aqueous two-phase extraction, integrated countercurrent chromatography). The results create a data-driven feedback to the USP, which will serve for media and process optimizations in order to reduce, or even eliminate nascent critical HCPs. This will improve separation efficiency and may lead to a quantitative process understanding. Different HCP species were classified by stringent criteria with regard to DSP separation parameters into “The Good, the Bad, and the Ugly” in terms of pI and MW using 2D-PAGE analysis depending on their positions on the gels. Those spots were identified using LC-MS/MS analysis. HCPs, which are especially difficult to remove and persistent throughout the DSP (i.e., “Bad” or “Ugly”), have to be evaluated by their ability to be separated. In this approach, HCPs, considered “Ugly,” represent proteins with a MW larger than 15 kDa and a pI between 7.30 and 9.30. “Bad” HCPs can likewise be classified using MW (>15 kDa) and pI (4.75–7.30 and 9.30–10.00). HCPs with a MW smaller than 15 kDa and a pI lower than 4.75 and higher than 10.00 are classified as “Good” since their physicochemical properties differ significantly from the product. In order to evaluate this classification scheme, it is of utmost importance to use orthogonal analytical methods such as IEX, HIC, and SEC.

## 1. Introduction

The amounts of biotechnology products produced worldwide, prescription as well as over-the-counter drugs, are estimated to account for around 50% of the most successful pharmaceutical products by the year 2020 [1]. Oncology constitutes the biggest therapeutic sector, with an annual growth rate of around 12.5% and sales of approximately $83.2 billion in 2015. Among the five top-selling oncological products, three will be monoclonal antibodies by the year 2020 [2]. The manufacturing process of biopharmaceuticals such as monoclonal antibodies (e.g., IgG, immunoglobulin G) is divided into upstream (USP) and downstream processing (DSP) [3,4,5,6,7,8]. The production of the monoclonal antibody in bioreactors (BR) using mammalian cells as an expression host and the separation of the liquid phase from the cells using centrifuges or filters is defined as USP [9]. The subsequent DSP is designed to separate side components like host cell proteins (HCP) or host cell DNA (hDNA) from the main component [6,7,10]. The most common unit operations used in the DSP are typically chromatography and filtration.

The commercial success of monoclonal antibodies of course led to significantly increased demand in their production scale [11]. Coping with these demands without significantly changing the approved manufacturing facilities almost forced companies to follow the route of increasing titers within the existing facilities.

Hence, compared to earlier yields of a couple of grams per liter, today antibody concentrations of up to 25 g/L can be achieved using a modified perfusion process [12,13]. Routinely, antibody concentrations of between three and five grams per liter can be generated in fed-batch processes [3,14,15]. However, increasing product titers at constant volumes due to higher cell concentrations will lead to capacity limitations in the DSP, which has to be compensated for by longer process times, higher material consumption, and corresponding costs [4]. This will significantly shift the cost of goods from the USP to the DSP [5]. Therefore, DSP technologies are required that circumvent this upcoming “downstream bottleneck,” handling high titer volumes [4,16,17]. 

**Figure 1:**
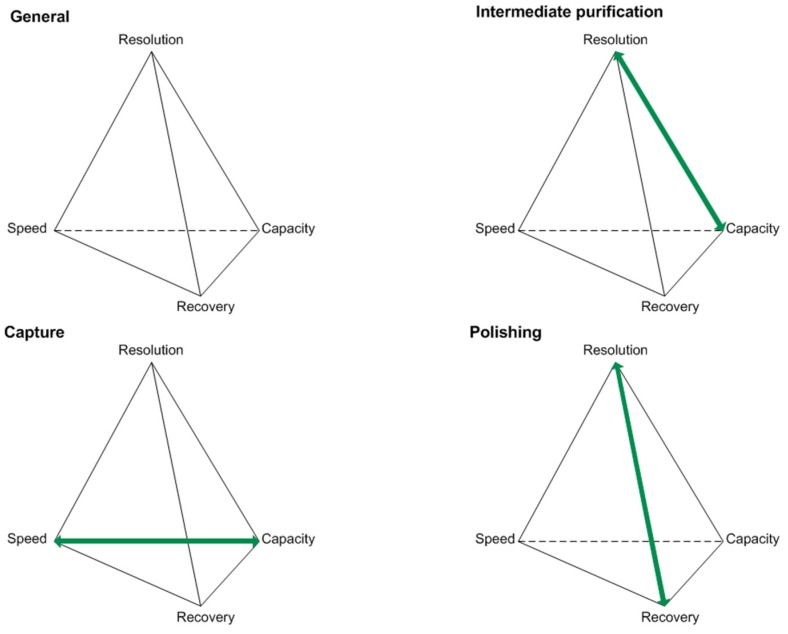
Key performance parameters of the capture, intermediate purification, and polishing step for protein purifications according to [18].

Optimizations in the USP concepts have led to increasing product titers. Along with this, raised impurity profiles have been observed [8,9]. Various compositions of the cultivation broth present challenges in the DSP of biotechnologically produced proteins. Considering the generic platform production process for antibodies, unit operations like centrifugation, micro- and ultrafiltration, protein A affinity chromatography, two orthogonal virus inactivation steps, ion-exchange (IEX), and hydrophobic interaction chromatography (HIC) are being used [5]. For the characterization of protein purification stages, key performance parameters can be used. These are typically resolution, speed, recovery, and capacity, as seen in Figure 1. 

The objectives vary depending on the purification stage in focus, and therefore generate different challenges that have to be addressed during process optimization [18]. For example, the protein A affinity chromatography, used as a capture step, will reach its capacity limitation due to increasing product titers. This can be problematic since this criterion characterizes the capture step and is one of its two objectives. Moving downstream, selectivity challenges occurring during the intermediate purification and polishing step will prevent each step from reaching one of its objectives. Selectivity challenges are going to affect the resolution in IEX and HIC separation operations when HCP resemble the product in terms of pI and hydrophobicity, respectively [12,18].

Impurities like HCPs, which resemble the desired product only in one characteristic (e.g., pI), may challenge the IEX but can probably be easily separated from the product by an additional chromatographic step (e.g., HIC). Impurities similar to the product in more than one characteristic (e.g., pI and hydrophobicity) will be troublesome during purification and polishing. Therefore, the pI and hydrophobic distribution of the impurity spectrum can negatively affect IEX and HIC separations, respectively. 

Critical performance parameters regarding the separation efficiency during the capture of monoclonal antibodies using affinity chromatography are capacity limitations as well as (un-)specific HCP co-elution [19]. Consequently, new approaches and technology are needed in order to circumvent future bottlenecks and separation challenges [4]. 

Furthermore, the existing challenges in process engineering have worsened since regulatory agencies demand higher product quality, an advanced understanding of the process and product, as well as batch-independent product quality [20,21,22]. Bioprocess engineering will probably focus in regulated industries on quality by design and process analytical technology mechanisms, in order to design, analyze, and control manufacturing processes [23]. This shall lead to improved process control by knowledge-based and statistical methods, which ultimately guarantees the process’ robustness.

**Figure 2:**
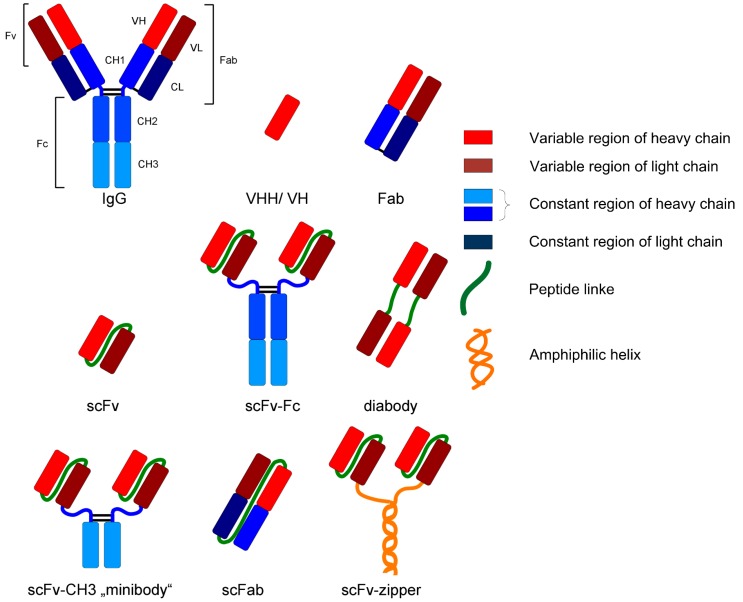
Various formats of recombinant antibodies [24].

For example, monoclonal antibodies and fragments represent an interesting group of biopharmaceuticals due to their broad field of application (e.g., analysis or diagnostic). Those glycoproteins are structurally complex and differ in various formats, as can be seen in Figure 2. IgG is the most common format as a biopharmaceutical drug [2].

**Figure 3:**
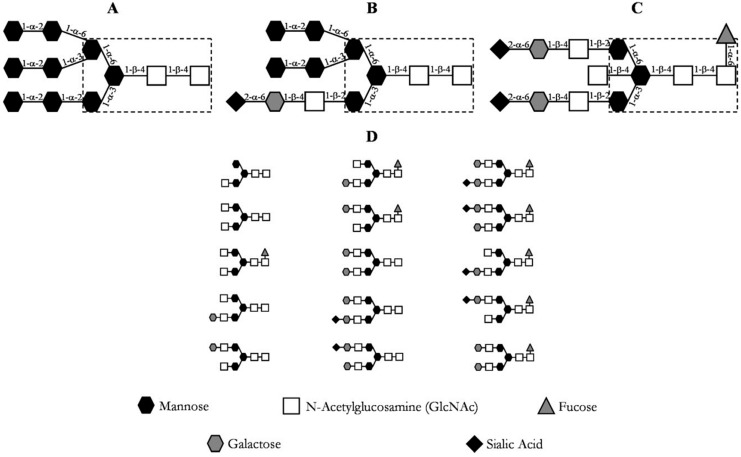
Common glycosylation patterns of an IgG. (**A**) high mannose content; (**B**) hybrid; (**C**) complex biantennary oligosaccharide with core fucosylation; (**D**) most prevalent oligosaccharide structures of IgG [20].

The post-translational modifications, especially glycosylations, of these proteins are of utmost importance for their correct function [20]. The immense diversity of glycosylation patterns impacts the functionality, immunogenicity, and pharmacokinetics of the antibody [24,25]. Due to this, posttranslational modifications should be considered critical quality attributes (CQA) and verified throughout the manufacturing of monoclonal antibodies [26,27,28,29,30]. Antibody N-glycans can be quantitatively determined by normal phase chromatography after N-glycosidase digestion and glycan labeling, for example [31]. The most prevalent N-linked glycosylation patterns at the Cγ2 domain of the heavy chain (Fc) of an immunoglobulin G (IgG) are depicted in Figure 3, where the most common glycosylation of IgG is shown in section D.

Besides critical process parameters (CPP) like pH, pO_2_ and pCO_2_, more often impurities play an important role in affecting CQA of the biopharmaceutical product. For example, extracellular proteases and glycosidases accumulating during the cultivation negatively influence the CQA of monoclonal antibodies [14,32,33,34,35]. The impurity spectrum consists of a multiplicity of different substances (HCP, hDNA, virus, cells, and cell debris). In this integration approach, HCPs are considered as the primary impurity based on their broad composition and range of isoelectric point (pI), molecular weight (MW), and hydrophobicity, as can be seen in Table 1 [36,37,38,39,40]. They exhibit no constant level, composition, or property distribution. HCPs caused by secretion or cell lysis can range in pI (2–11), MW (10–200 kDa), and variable hydrophobicity, and are therefore difficult to separate if their physicochemical properties resemble the product of interest.

Primary recovery and purification steps for a biopharmaceutical DSP are based on physicochemical properties in order to efficiently purify the product. However, especially in the case of increasing product titers, a sub-population of impurities (i.e., HCP), which negatively affect the product quality, may remain with the desired protein and represent a certain risk [40]. Therefore, it is of critical importance to validate qualitatively and quantitatively the separation efficiency of each unit operation in the DSP. 

This assessment will lead to an expanded understanding of each unit operation by classifying the impurities into “The Good, the Bad, and the Ugly”:

- Impurities, which can be separated easily from the main component, are considered “the Good.” They possess physicochemical properties significantly different from the protein of interest (i.e., pI, MW, hydrophobicity). As a result, they may be separated by only one unit operation in an efficient way (ion exchange in terms of charge differences).
- Side components showing more similarity to the product are more difficult to separate or are persistent throughout (i.e., not separable from the product) and thus are considered as “the Bad” or “the Ugly.”

**Figure 4:**
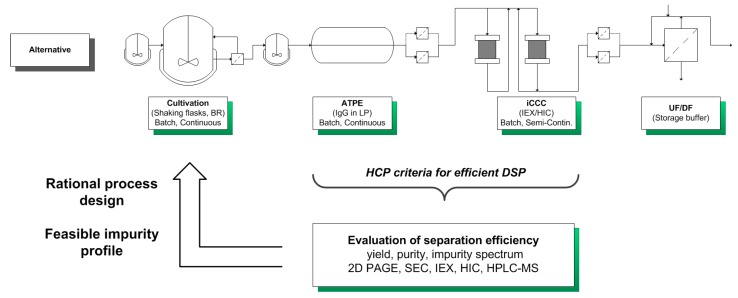
USP DSP integration approach for a systematic development of a bioprocess.

By characterizing the HCP criteria for an efficient DSP, it is possible to gain a deeper understanding of the process and preserve the quality of the product. This categorization can be used for an USP DSP integration approach towards an efficient production process by circumventing the generation or accumulation of “Bad” and “Ugly” impurities (Figure 4). 

The considered process for the production of monoclonal antibodies utilizes mammalian cell cultivations. Afterwards, the aqueous two-phase extraction (ATPE) is used as a cell harvesting or capture step, depending on the system composition used [41,42,43,44,45]. Following the ATPE, the integrated counter current chromatography (iCCC), which is a combination of an IEX and HIC, is employed as a purification and polishing step. This combination of chromatographic columns leads to a highly purified product [46]. 

The integration approach begins with a data-driven characterization of HCP occurring in the broth and in each unit operation. The separation efficiency is determined by analytical methods (i.e., 2D SDS-PAGE, SEC, IEX, HIC, and HPLC-MS/MS). The SEC chromatograms qualitatively describe the impurity spectrum and can be used for a determination of impurities in the molecular weight range of the considered product (150 kDa). The IEX and HIC are used for characterizing the charge and hydrophobicity of the HCPs. 2D SDS-PAGE analysis, combined with HPLC-MS/MS measurements, is used for the identification and, of utmost importance, classification of “The Good, the Bad, and the Ugly” HCPs. This classification is done by evaluating the molecular weight, isoelectric point, and hydrophobicity of the HCPs, as seen in Table 2.

Afterwards, these findings are used in rational process design in order to minimize or even eliminate “Ugly” HCPs, which cannot be easily separated from the product (Figure 4).

One possible process design optimization procedure is the improvement of media components. Media optimization is capable of changing the broth’s HCP composition towards a population that is easier to separate or at least exhibits a lower HCP concentration. In addition, an optimized medium not only shifts the HCP profile but also improves the cell growth and product titer, which is depicted in Table 3 [47].

**Figure 5:**
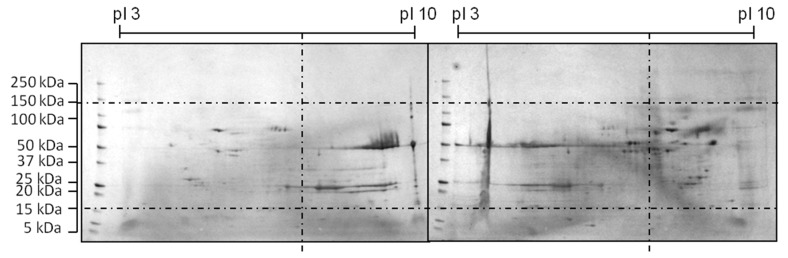
Comparison of 2D-SDS PAGE of a reference (**right**) and optimized medium (**left**) during a CHO cultivation according to [47]. Media was improved by a three-level DoE design.

The shifted HCP profile can be seen in the 2D-SDS PAGE comparison in Figure 5.

In this work, the results of the characterization of the HCP profile from a mAb production process are presented. Process-related data as well as analysis-related data are used for the characterization of the process and for the classification of HCPs. The results of each analytical method are critically evaluated in order to determine a process flow being suitable for USP DSP integration and process optimization. Analytical methods such as SEC, 2D-PAGE, IEX, HIC as well as HPLC-MS/MS were used in order to identify critical HCP in the cell-free broth and during each unit operation (i.e., ATPE, IEX, and HIC).

## 2. Results and Discussion

**Figure 6:**
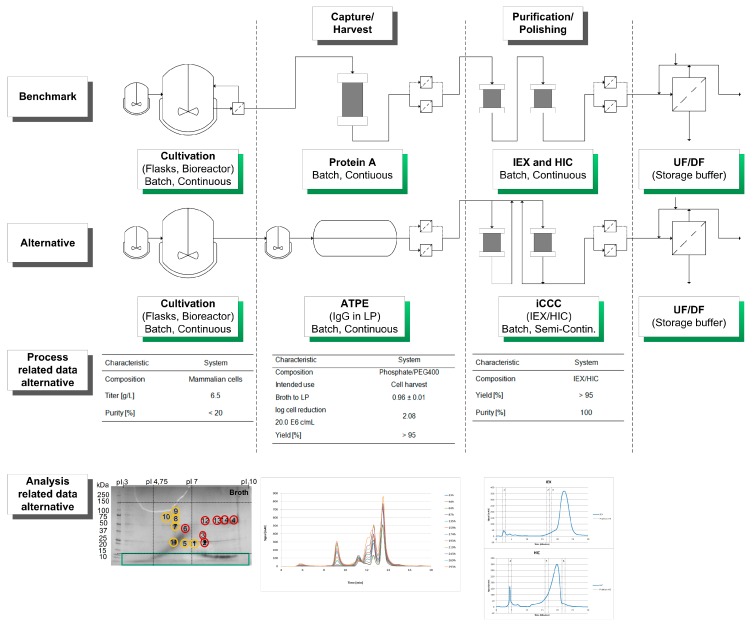
Schematic overview of the considered alternative process in comparison of the benchmark manufacturing route. In addition, process and analysis related data are shown and discussed in the text.

A schematic overview of the considered alternative process as well as process- and analysis-related data are shown in Figure 6.

The HCP criteria for an efficient DSP have to be evaluated for each unit operation, according to Figure 4. Here, the classification of HCP focuses on the broth, the broth after diafiltration and on a side component fraction after HIC separation. Process-related data such as titer, yield, and purity of each unit operation are shown in Table 4.

This analytical procedure focuses on the classification and characterization of HCPs. Therefore, each unit operation of the DSP has to be evaluated by its separation efficiency, using analytical methods such as 2D-SDS PAGE, IEX, HIC, and SEC to determine HCP criteria for an efficient DSP, as seen in Figure 3. Protein A and size-exclusion chromatography are used to determine yield and purity, respectively. Each unit operation was loaded with the native broth in order to determine their separation efficiency.

**Figure 7:**
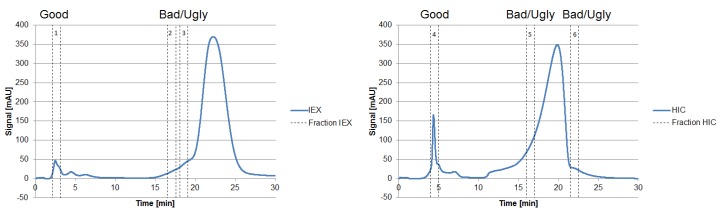
Chromatograms of an analytical IEX (**left**) and HIC (**right**) measurement of the diafiltrated cell-free CHO supernatant. The vertical sections represent the number of fractions taken, representing “Good”, “Bad,” and “Ugly” impurities.

**Figure 8:**
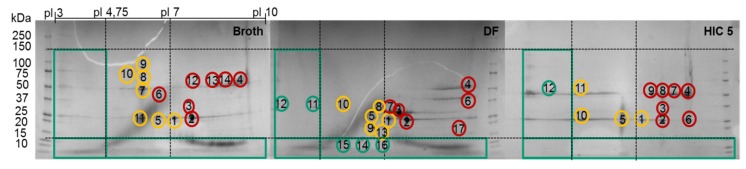
2D SDS-PAGE of the broth, diafiltrated broth (DF) and HIC fraction. Green circles represent “Good”, yellow circles “Bad,” and red circles “Ugly” HCP.

The fraction number five occurring on the HIC was chosen due to the high side component content near the target product, as seen in Figure 7. In the following, the classification of the HCPs will be performed by 2D-PAGE gels, as depicted in Figure 8. 

The classification criterion of the considered HCP was selected by comparing their pI and molecular weight to the target product, as seen in Table 5.

While considering the 2D-PAGE gels, proteins with a MW lower than 15 kDa can be considered “Good” since they can be separated by using diafiltration subsequent to an ATPE with a suitable MW cutoff. Therefore, this filtration step is coupled to a buffer change, which is necessary for the use of the iCCC, since the specific light phase contains PEG400, resulting in a more viscous solution, which would make the chromatographic steps more difficult to handle.

**Figure 9:**
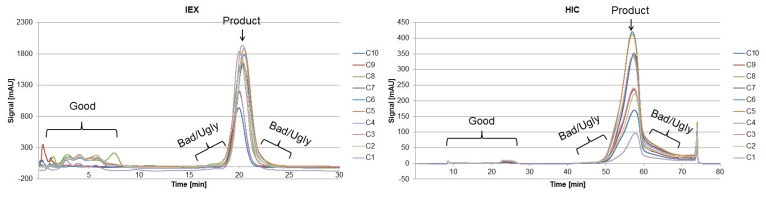
Chromatograms of the IEX (**left**) and HIC (**right**) after various cycles in the iCCC mode.

Proteins larger than 15 kDa have to be separated by another unit operation, which is based on other physicochemical properties (i.e., pI, hydrophobicity). Therefore, “Bad” and “Ugly” proteins possess a MW larger than 15 kDa. The horizontal line at 150 kDa represents the target protein in its functional condition. The vertical lines depict the isoelectric point at 4.75 and 7.0. Impurities with a pI of 4.75 can be subjected to a possible precipitation step using hydrochloric acid, which significantly reduces their concentration [42]. Those impurities exhibit a different pI than the target protein and can efficiently be separated by an IEX and are therefore classified as “Good.” Experimental IEX data show a distinctly different interaction with the stationary phase due to their surface charge distribution, as seen in Figure 9, which resemble the “Good” HCP. They elute near the void volume and can be easily separated. A similar train of thought can be conducted while characterizing the HIC chromatogram. As can be seen in Figure 9, the target product gets concentrated by each cycle in the iCCC mode.

Impurities with a pI range close to the target protein (i.e., 7.30–9.30) are more difficult to separate via an IEX and are therefore considered “Ugly.” However, since the separation efficiency of the IEX will depend on the column, buffer solution, and process parameters, this range can vary depending on the system used. Impurities with characteristics in between those of “Good” and “Ugly” are defined as “Bad.” They possess a pI of 4.75–7.30 and 9.30–10.00 and can be difficult to separate when other physicochemical properties (i.e., hydrophobicity) resemble the target product. This dependency can of course also occur with “Ugly” HCP but since they are already classified as difficult to separate, they will not be characterized differently. Regarding the “Good” HCP, this dependency will not occur even if other physicochemical properties show close similarity to the product, since at least one physicochemical attribute is significantly different from the target product. The pI is restricted to 10 due to the pH gradient used in the IEF prior to 2D gel electrophoresis.

**Figure 10:**
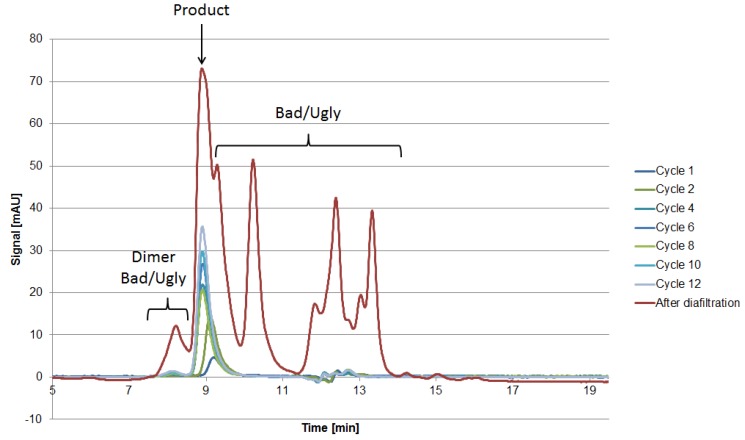
SEC chromatograms after various cycles as well as the broth after diafiltration.

The 2D-PAGE analysis seen in Figure 8 is suitable for the visualization of the side component spectrum. However, the sample preparation requires reducing agents such as DTT, which destroys the protein’s structure by reducing the disulfide bonds. This preparation procedure results in spots on the gel, which do not resemble their native structure in the supernatant. Following the aforementioned classification and separation system, proteins with a MW lower than 15 kDa but with an isoelectric point near the target product will sometimes be classified as “Good” since they can be separated by filtration. Hence, the native protein can be “Ugly” even if it appears as “Good” in the gel (assuming no change in surface charge). Thus, it is of the utmost importance to use orthogonal analytical methods to validate the classification. In terms of MW, SEC analysis can be conducted in order to determine the size distribution of side components, as seen in Figure 10. The advantage of using SEC analysis is the determination of the side component’s native MW distribution as well as their qualitative mass in proportion to the product’s signal. The disadvantage is the less sensitive detection of low mass content side components as well as proteins resulting in a signal overlap with the mAb.

In order to identify proteins present in the 2D-PAGE gel spots, their tryptic peptides were identified via analysis with liquid chromatography (LC) coupled to tandem mass spectrometry (MS/MS) and a database search. The numbers in the gels in Figure 8 indicate the spots that were analyzed using LC-MS/MS. The first five spots occurred in every gel. The subsequent spots were unique in each gel. The identified peptides and their corresponding proteins of each spot in these gels are presented in the appendix (Table A1, Table A2 and Table A3). The identified proteins of the first recurrent spots are listed alongside with their MW and pI in Table 6.

As can be seen in Table 6, the MW and pI of the spots analyzed with 2D-PAGE do not correspond to the value in the protein database. This is a result of proteins existing in different species due to posttranslational modifications and proteolytic processing (proteolytic degradation and sample preparation, respectively). In contrast, the theoretically calculated pI values obtained by the ExPASy computation tool (http://web.expasy.org/compute_pi/) represent the unmodified full length amino acid sequence of a defined protein. SEC analysis, for example, is a non-invasive analytical method for the determination of MW distribution of side components, if the salt concentration used in aqueous eluents allows for separation based on molecular size exclusion alone due to the hydrodynamic radius [48]. Nevertheless, for a systematic integration approach, the classification of HCPs based on their physicochemical properties can lead to an enhanced process understanding, especially in the DSP.

## 3. Materials and Methods

Chinese hamster ovary cells (CHO DG44) were used for the production of a monoclonal antibody. The culture conditions were 37 °C, 5% carbon dioxide, and 130 rpm. The cultivations were carried out in shake flasks in a serum-free medium.

The ATP system applied consisted of 44.5% broth, 15.5% PEG400 (Merck KGaA, Darmstadt, Germany), and 40% of a 40 wt% phosphate buffer. All the components were weighed. The extraction was carried out at pH 6.0 in 50-mL beakers at room temperature. The system was mixed for 15 min at 140 rpm in an incubator shaker. Phase separation took place within 30 min in a separatory funnel. 

The broth was diafiltrated using a SARTOFLOW^®^ Slice 200 Benchtop system from Sartorius Stedim (Germany). A 10 kDa Hydrosart^®^ (Sartorius Stedim, Göttingen, Germany) was utilized as a membrane module. 

The iCCC (integrated counter-current chromatography) is run by using Fractogel^®^ EMD SO_3_^−^(s) and Fractogel^®^ EMD Phenyl(s) (Merck KGaA, Darmstadt, Germany). The buffers consisted of a 20 mM sodium phosphate buffer (Na_2_HPO_4_, NaH_2_PO_4_) as well as a 20 mM sodium phosphate buffer with 1 M Na_2_SO_4_. 

The product was quantified by Protein A chromatography (PA ID Sensor Cartridge, Applied Biosystems, Bedford, MA, USA). Dulbecco’s PBS buffer (Sigma-Aldrich, St. Louis, MO, USA) was used as a loading buffer at pH 7.4 and as an elution buffer at pH 2.6. The absorbance was monitored at 280 nm. 

The size exclusion chromatography was done by using a Yarra™ 3 µm SEC-3000 column (Phenomenex Ltd., Aschaffenburg, Germany) with 0.1 M Na_2_SO_4_, 0.1 M Na_2_HPO_4_, and 0.1 M NaH_2_PO_4_ (Merck KGaA, Germany) as a buffer system. 

Isoelectric focusing was carried out using IPG strips (ReadyStripTM IPG Strips, linear, pH 3–10, BIO-RAD, Hercules, CA, USA) and an isoelectric focusing unit of Hoefer (Hoefer Inc., Holliston, MA, USA). A subsequent SDS PAGE was carried out using gels (Criterion TGX Precast Gel, 4–15% Bis-Tris, BIO-RAD), buffers, and an electrophoresis chamber from BIO-RAD. The resulting gels were colored by Coomassie Brilliant Blue G-250 (VWR International, Radnor, PA, USA).

For the identification of proteins, selected 2D GE spots were cut out and reduced into 1-mm^2^ pieces. After reduction of the disulfide bonds with 10 mM dl-dithiothreitol (Sigma-Aldrich) and alkylation with 50 mM iodoacetamide (Sigma-Aldrich), an in-gel proteolytic digestion was performed with 8 ng/µL trypsin (Promega, Madison, WI, USA) at 37 °C overnight. The peptides were extracted from the gel with 65% acetonitrile and 5% acetic acid in water and the solvent was evaporated to complete dryness. The peptides were re-suspended in 20 µL 0.1% formic acid (Fluka) and subjected to LC-MS/MS analysis with a nano-flow ultra-performance liquid chromatography (nano-UPLC) system (nanoACQUITY, Waters, Manchester, UK) coupled via an electrospray-ionization (ESI) source to a tandem mass spectrometer (MS/MS) consisting of a quadrupole and a orbitrap mass analyzer (Orbitrap QExcactive, Thermo Scientific, Bremen, Germany). Four microliters of each sample were loaded onto a reversed-phase (RP) trapping column (Symetry C18 Trap Column; 100 Å, 5 µm, 180 µm × 20 mm) and washed with 1% buffer B for 5 min. The peptides were eluted onto a RP capillary column (nanoAcquity Peptide BEH analytical column; 130 Å, 1.7 µm, 75 µm × 200 mm) and separated by a gradient from 3 to 35% buffer B in 35 min (250 nL/min). Eluting peptides were ionized and desorbed by ESI in the positive mode using a fused-silica emitter (I.D. 10 μm, New Objective, Woburn, MA, USA) at a capillary voltage of 1800 V. Data-dependent acquisition mode was used with the following parameters: MS level over a *m*/*z* range from 400 to 1500, with a resolution of 70,000 FWHM at *m*/*z* 200. Maximum injection time was set to 120 ms for an AGC target of 1E6. For MS/MS analysis the top 12 signals were isolated in a 2 *m*/*z* window and fragmented with a normalized HCD collision energy of 25. Fragment spectra were recorded with a resolution of 17,500 FWHM at *m*/*z* 200. Maximum injection time was set to 60 ms for an AGC target 5E5.

LC–MS raw data were processed with MaxQuant (Max Planck Institute of Biochemistry, Planegg, Germany) algorithms (version 1.5.8.3). Protein identification was carried out with Andromeda against a hamster (*Cricetulus griseus*) (www.uniprot.org, downloaded on 31 January 2017) and a contaminant database. The searches were performed using a precursor mass tolerance set to 10 ppm and fragment mass tolerance set to 20 ppm. For peptide identification, two missed cleavages were allowed, a carbamidomethylation on the cysteine as a fixed modification and oxidation of the methionine as a variable modification. A maximum of five modifications per peptide were allowed.

## 4. Conclusions

The presented approach of integrating USP and DSP is based on the classification and characterization of impurities generated during USP. This will lead to a deeper quantitative process understanding and identification of issues in the DSP early on. Here, the HCPs were categorized into “The Good, the Bad, and the Ugly” by evaluating their physicochemical properties compared to the monoclonal antibody. In this approach “Good” impurities possess a MW lower than 15 kDa and a pI lower than 4.75. “Ugly” impurities on the other hand exhibit a pI of 7.3–9.3, whereas “Bad” impurities feature a pI between 4.75 and 7.3 as well as between 9.3 and 10.0. In order to evaluate the classification system for the generated HCPs, orthogonal analytical methods are of utmost importance. IEX and SEC analysis were conducted for the identification of impurities. Theoretical pI and MW calculated based on the amino acid sequence differ from the experimental values obtained in 2D gel electrophoresis. This is due to not considering posttranslational modifications, as well as in vivo and ex vivo proteolytic processing. 

Nevertheless, it is possible to characterize HCP based on pI and MW properties. In order to fully categorize the separation efficiency of each unit operation in the DSP as well as of their combinations, the HCP profile has to be determined with the aforementioned analytical methods in future approaches. This portfolio can of course be extended by adding supplementary methods like NMR technologies, preferably online [49]. 

Considering the significant amount of work in terms of characterization, monitoring, and removal of impurities and contaminations created by the USP step, as well as the time and cost associated with their removal, it may be worthwhile to reflect in more detail how these impurities and product variations are generated in the first place. Work to this end already started some time ago. Initial results and corresponding concepts for a more balanced integrated process design will be presented in the near future. 

## Figures and Tables

**Table 1:** Physicochemical properties of the main impurities during the production of biopharmaceuticals, according to [38].

Class	pI	MW (kDa)	Hydrophobicity	Origin	Cause
HCP	2–11	10–200	Variable	Host cells	Secretion, lysis
hDNA	2–3	90–1000	Low	Host cells	Lysis
Insulin	5.3–5.5	5.8	Low	Media	Supplement
Virus	4–7.5	200–7200	Variable	Host cells, media	Contamination
Endotoxins	1–4	3–40	Variable	Media, contamination	Contamination

**Table 2:** Analytical methods used for the characterization of HCP.

Characteristic	Method	Orthogonal Method
Isoelectric point	2D-SDS PAGE	IEX; HPLC-MS/MS
Molecular weight	SEC	2D-SDS PAGE; HPLC-MS/MS
Hydrophobicity	HIC	-

**Table 3:** Improved parameters by using an optimized medium according to [47].

Parameter	Optimized medium
Titer increase	Factor 2.5
Cell growth	Factor 2–2.3
IgG/HCP	65%
HCP profile	Shift

**Table 4:** Process related data of the cultivation, ATPE, and iCCC. Yield and purity were determined using protein A chromatography and SEC, respectively.

	Cultivation	ATPE	iCCC
System	Mammalian cells	PEG400/40 wt% PO_4_	IEX/HIC combination
Titer/yield	6.5 g/L	>95%	>95%
Log cell reduction 20.0 E6 cells/mL	-	2.08	-
Purity	<20%	up to 80% *	100%

* Protein-based according to SEC.

**Table 5:** Classification of the “Good, Bad, and Ugly” HCP in comparison to the physicochemical properties of the monoclonal antibody (mAb). MW, molecular weight; pI, isoelectric point.

Characteristic	mAb	Good	Bad	Ugly
MW [kDa]	144.2	<15	>15	>15
pI [−]	8.30	<4.75 >10.00	4.75–7.30 9.30–10.00	7.30–9.30

**Table 6:** Classification of CHO proteins identified via LC-MS/MS analysis and characterized by 2D-PAGE gel. Comparison of theoretical (UniProt; pI calculated according to the amino acid sequences) and observed MW and pI with respect to the spot position on the 2D-PAGE gel.

Spot Gel	MW Gel	pI Gel	Class Gel	MW ^1^	pI ^2^	Class MS	Protein	UniProt Accession Number
1	25	7.0	Bad	81.56	5.69	Bad	Glutathione S-transferase Mu7-like protein	A0A061IN16
2	25	7.5	Ugly	102.7	6.02	Bad	Actin, cytoplasmic 1	A0A069C7Y3
3	30	7.6	Ugly	38.03	6.08	Bad	Purine nucleoside phosphorylase-like protein	A0A061ILE8
4	50	9.4	Bad	72.13	7.23	Ugly	Pyruvate kinase	A0A098KXF7
5	25	6.3	Bad	38.03	6.08	Bad	Purine nucleoside phosphorylase-like protein	A0A061ILE8

^1^ Theoretical values according to the unmodified full length protein according to UniProt; ^2^ Theoretical values calculated using the ExPASy computation tool (http://web.expasy.org/compute_pi/).

## Appendix A

**Table A1:** Peptides and their corresponding proteins identified via LC-MS/MS in spots of 2D gel of the broth. Molecular weight (MW) and isoelectric point (pI) of the unmodified full length protein according to UniProt.

#	Gel MW	Gel pI	MW (UniProt)	pI (UniProt)	Primary Accession Number (UniProt)	Number of Unique Peptides	Protein
1	25	7.2	25.76	6.45	A0A061HUZ2	9	Platelet-activating factor
			81.56	5.69	A0A061IN16	11	Glutathione S-transferase Mu 7-like protein
			25.88	6.43	A0A061HYZ1	9	Peroxiredoxin-6-like protein
2	25	7.9	30.28	7.22	A0A061IFC9	4	Carbonic anhydrase
			89.52	6.23	A0A061IJC4	4	Glutathione S-transferase Mu 1-like protein
			81.56	5.69	A0A061IN16	4	Glutathione S-transferase Mu 7-like protein
3	30	7.6	72.13	7.23	A0A098KXF7	8	Pyruvate kinase
			38.03	6.08	A0A061ILE8	6	Purine nucleoside phosphorylase-like protein
			32.23	9.11	A0A061IAK4	5	l-lactate dehydrogenase A chain
4	50	9.6	45.28	8.48	A0A061IB69	7	Fructose-bisphosphate aldolase
			72.13	7.23	A0A098KXF7	15	Pyruvate kinase
			102.7	6.02	A0A069C7Y3	5	Actin, cytoplasmic 1
5	25	6.6	27.39	6.34	A0A061I2E1	8	Proteasome subunit
			89.52	6.23	A0A061IJC4	8	Glutathione S-transferase Mu 1-like protein
			81.56	5.69	A0A061IN16	8	Glutathione S-transferase Mu 7-like protein
6	45	6.7	50.57	5.93	G3GR73	11	Rab GDP diss. inhib.
			52.79	6	A0A098KXB1	10	Cytosol aminopeptidase-like protein
			44.67	7.54	A0A061IJI8	9	Alpha-enolase
7	50	6.1	52.79	6	A0A098KXB1	20	Aminopeptidase
			72.13	7.23	A0A098KXF7	29	Pyruvate kinase
			145.1	8.37	A0A061HU29	15	Glucose-6-phosphate 1-dehydrogenase
8	57	6	73.86	5.56	A0A061I5D1	22	Heat shock protein
			74.72	5.29	A0A061HWC7	9	Plastin-3
			69.64	5.57	A0A061I5U1	9	Heat shock-related protein 2
9	80	6.1	72.13	7.23	A0A098KXF7	11	Pyruvate kinase
			117.7	5.42	G3IBG3	8	Ubiquitin activating enzyme E1
			73.86	5.56	A0A061I5D1	7	Heat shock protein
10	70	5.6	73.86	5.56	A0A061I5D1	9	Heat shock protein
			68.43	5.55	A0A061I1Q2	5	Vitamin K-dependent protein S
			85.71	5.2	A0A061IAX6	5	Dipeptidyl peptidase 3
11	25	6	25.88	6.43	A0A061HYZ1	9	Peroxiredoxin
			89.52	6.23	A0A061IJC4	19	Glutathione S-transferase Mu 1-like protein
			81.56	5.69	A0A061IN16	14	Glutathione S-transferase Mu 7-like protein
12	55	7.8	72.13	7.23	A0A098KXF7	37	Pyruvate kinase
			52.79	6	A0A098KXB1	10	Cytosol aminopeptidase-like protein
			73.86	5.56	A0A061I5D1	4	Heat shock protein
13	50	8.6	52.79	6	A0A098KXB1	4	Cytosol aminopeptidase-like protein
			72.13	7.23	A0A098KXF7	19	Pyruvate kinase
			145.1	8.37	A0A061HU29	2	Glucose-6-phosphate 1-dehydrogenase
14	50	9.2	72.13	7.23	A0A098KXF7	20	Pyruvate kinase
			44.67	7.54	A0A061IJI8	3	Alpha-enolase
			42.69	8.78	A0A061HV36	3	Eukaryotic translation initiation factor 2 subunit 3-like protein

**Table A2:** Peptides and their corresponding proteins identified via LC-MS/MS in spots of 2D gel of the diafiltrated broth. Molecular weight (MW) and isoelectric point (pI) of the unmodified full length protein according to UniProt.

#	Gel MW	Gel pI	MW (UniProt)	pI (UniProt)	Primary accession number (UniProt)	Number of Unique Peptides	Protein
1	25	7	26.96	5.38	A0A061I6A0	5	Glutathione S-transferase A4-like protein
			81.56	5.69	A0A061IN16	8	Glutathione S-transferase Mu 7-like protein
			38.03	6.08	A0A061ILE8	8	Purine nucleoside phosphorylase-like protein
2	25	7.5	38.03	6.08	A0A061ILE8	6	Purine nucleoside phosphorylase
			102.7	6.02	A0A069C7Y3	2	Actin, cytoplasmic 1
			43.35	6.48	A0A061IJG8	2	Prostaglandin reductase 1-like protein
3	30	7.4	45.28	8.48	A0A061IB69	1	Fructose-bisphosphate aldolase
4	50	9.4	89.52	6.23	A0A061IJC4	2	Glutathione S-transferase
5	25	6.6	38.03	6.08	A0A061ILE8	7	Purine nucleoside phosphorylase
			89.52	6.23	A0A061IJC4	5	Glutathione S-transferase
6	37	9.4	45.28	8.48	A0A061IB69	4	Fructose-bisphosphate aldolase
			43.35	6.48	A0A061IJG8	2	Prostaglandin reductase 1-like protein
			361.89	4.81	A0A061IH02	2	Desmoglein-4-like protein
7	30	7	38.03	6.08	A0A061ILE8	4	Purine nucleoside phosphorylase
			128.68	6.78	A0A061IK77	3	Exosome component 10 isoform 1
			11.37	11.36	G3H2T6	2	Histone H4
8	30	6.8	27.79	4.7	A0A061IGS6	4	Protein sigma
			102.7	6.02	A0A069C7Y3	5	Actin, cytoplasmic 1
			361.89	4.81	A0A061IH02	4	Desmoglein-4-like protein
9	17	6.6	11.37	11.36	G3H2T6	4	Histone H4
			14.99	10.2	A0A061IP52	2	Histone H2B
10	30	5.6	52.25	5.35	A0A061IML2	13	Annexin
			268.7	5.69	A0A061IP39	10	Filamin-B isoform 4
			50.99	6.94	A0A061I8I4	4	Cathepsin F
11	30	4.5	52.25	5.35	A0A061IML2	2	Annexin
			14.73	9.87	A0A061IQB8	3	Ubiquitin-60S
12	30	2.8	38.03	6.08	A0A061ILE8	8	Purine nucleoside phosphorylase
			89.52	6.23	A0A061IJC4	8	Glutathione S-transferase
			101.51	5.12	A0A061IRD9	5	AP complex subunit beta
13	15	6.7	38.03	6.08	A0A061ILE8	4	Purine nucleoside phosphorylase
			89.52	6.23	A0A061IJC4	4	Glutathione S-transferase
			38.31	5.33	A0A061IEW1	3	Nuclear migration protein nudC-like protein
14	15	6.1	38.31	5.33	A0A061IEW1	5	Nuclear migration protein nudC-like protein
			89.52	6.23	A0A061IJC4	4	Glutathione S-transferase
			54.11	5.01	A0A061IDB2	3	Prelamin-A/C-like isoform 1
15	12	5.6	17.16	7.8	A0A061I0I3	4	SH3 binding protein
			17.19	5.94	G3HBD4	3	Nucleoside diphosphate kinase
16	17	6.6	23.42	5.1	G3GXB0	3	Rho GDP
			89.52	6.23	A0A061IJC4	8	Glutathione S-transferase
			102.7	6.02	A0A069C7Y3	2	Actin, cytoplasmic 1
17	16	9.2	14.73	9.87	A0A061IQB8	3	Ubiquitin-60S
			102.7	6.02	A0A069C7Y3	3	Actin, cytoplasmic 1
			23.42	5.1	G3GXB0	3	Rho GDP

**Table A3:** Peptides and their corresponding proteins identified via LC-MS/MS in spots of 2D gel of the HIC fraction. Molecular weight (MW) and isoelectric point (pI) of the unmodified full length protein according to UniProt.

#	Gel MW	Gel pI	MW (UniProt)	pI (UniProt)	Primary Accession Number (UniProt)	Number of Unique Peptides	Protein
1	25	6.9	-	-	-	-	-
2	25	7.6	11.37	11.36	G3H2T6	2	Histone H4
			102.7	6.02	A0A069C7Y3	2	Actin, cytoplasmic 1
3	30	7.6	38.03	6.08	A0A061ILE8	2	Purine nucleoside
			102.7	6.02	A0A069C7Y3	1	Actin, cytoplasmic 1
4	50	8.4	44.67	7.54	A0A061IJI8	8	Alpha-enolase
			72.13	7.23	A0A098KXF7	8	Pyruvate kinase
			38.03	6.08	A0A061ILE8	2	Purine nucleoside
5	25	6.3	38.03	6.08	A0A061ILE8	6	Purine nucleoside
6	25	8.4	38.03	6.08	A0A061ILE8	4	Purine nucleoside
7	50	8	44.67	7.54	A0A061IJI8	1	Alpha-enolase
8	47	7.6	44.67	7.54	A0A061IJI8	5	Alpha-enolase
			102.7	6.02	A0A069C7Y3	3	Actin, cytoplasmic 1
			72.13	7.23	A0A098KXF7	2	Pyruvate kinase
9	50	7.2	59.76	9.22	A0A061ICE4	4	ATP synthase subunit
			14.73	9.87	A0A061IQB8	2	Ubiquitin-60S ribosomal protein L40-like isoform 2
			102.7	6.02	A0A069C7Y3	2	Actin, cytoplasmic 1
10	25	5.1	-	-	-	-	-
11	50	4.8	211.66	5.42	A0A061I4N6	1	CAP-Gly domain-containing linker protein 1
12	52	4	-	-	-	-	-

(-) Spots, which were not able to be identified.
